# Inference of miRNA targets using evolutionary conservation and pathway analysis

**DOI:** 10.1186/1471-2105-8-248

**Published:** 2007-07-12

**Authors:** Dimos Gaidatzis, Erik van Nimwegen, Jean Hausser, Mihaela Zavolan

**Affiliations:** 1 Biozentrum, University of Basel, Basel, Switzerland

In our manuscript on miRNA target predictions [1] we made use of a number of genome alignments that we obtained from the UCSC genome browser. We regret that, due to a misunderstanding, we failed to explicitly acknowledge the sequencing centers that made the genome sequences, that were used to construct these alignments, available before publication.

These centers are: the Agencourt Bioscience Corporation [2] for the Drosophila ananassae, Drosophila mojavensis, and Drosophila virilis sequence data, the Genome Sequencing Center at the Washington University School of Medicine [3] for the Drosophila yakuba, Drosophila simulans, and Caernorhabditis remanei sequence data, the Human Genome Sequencing Center at the Baylor College of Medicine [4] for the Bos taurus and Rhesus macaque genome sequence, the Broad Institute [5] for the Monodelphis domestica genome sequence, and the Sanger Center [6] for the Danio rerio genome sequence.

We understand that continuing free access to data from large scale sequencing efforts requires careful acknowledgement and we apologize sincerely for this unfortunate oversight.
